# Precision Neutron Polarimetry for Neutron Beta Decay

**DOI:** 10.6028/jres.110.045

**Published:** 2005-06-01

**Authors:** S. I. Penttila, J. D. Bowman

**Affiliations:** Los Alamos National Laboratory Los Alamos, NM 87545

**Keywords:** neutron beta decay, neutron polarimetry, neutron time of flight, neutron polarization

## Abstract

The abBA collaboration is developing a new type of field-expansion spectrometer for a measurement of the three correlation coefficients *a*, *A*, and *B* and the shape parameter *b*. The measurement of *A* and *B* requires precision neutron polarimetry. We will polarize a pulsed cold neutron beam from the SNS using a ^3^He neutron spin filter. The well-known polarizing cross section for n-^3^He has a 1/*v* dependence, where *v* is the neutron velocity, which is used to determine the absolute beam polarization through a time-of-flight (TOF) measurement. We show that by measuring the TOF dependence of *A* and *B*, the coefficients and the neutron polarization can be determined with a small loss of the statistical precision and with negligible systematic error. We conclude that it is possible to determine the neutron polarization averaged over a long run in the neutron beta decay experiment with a statistical error less than 10^−4^. We discuss various sources of systematic uncertainty in the measurement of *A* and *B* and conclude that the fractional systematic errors are less than 2 × 10^−4^.

## 1. Introduction. A New Approach—*In Situ* Polarization Determination

Systematic uncertainties in the measurement of the neutron polarization introduce important systematic uncertainties in measurements of the neutron decay correlation coefficients *A* and *B*. The proposed abBA experiment [1] will polarize pulsed cold neutrons from the SNS by transmitting the neutron beam through a polarized ^3^He spin filter cell. An adiabatic radio-frequency spin flipper (RFSF) is used for a pulse-by-pulse neutron spin reversal. After the RFSF the neutrons are guided from the low magnetic field region, a few mT, into the decay volume of the spectrometer at 3.2 T. The pulse nature of the SNS beam and dependence of the neutron polarization on the neutron energy are used to determine accurately the beam polarization through a TOF measurement.

The beam polarization after the ^3^He spin filter is *P_n_* = tanh(*t*/*τ*), $\tau =\frac{L}{P_{3}\rho_{3}l\sigma_{1}\nu_{1}}$, *l* is the cell length, *σ*_1_ is the polarizing cross section at the neutron velocity *v*_1_, *L* is the flight path length, *ρ*_3_ in the number of ^3^He atoms per cm^3^, and *P*_3_ is the ^3^He polarization. The polarizing cross section has the well-known energy (velocity) dependence, $\sigma_{1}=848\frac{\sqrt{1\text{eV}}}{\sqrt{E}}$ barns. The decay spectrometer that detects coincidences between electrons and protons, measures simultaneously the beta decay asymmetries $\varepsilon_{B}=\frac{N_{p}↓-N_{p}↑}{N_{p}↓+N_{p}↑}=B\;\tanh(t/\tau )(1+\Delta )$ and $\varepsilon_{A}=\frac{N_{e}↑-N_{e}↓}{N_{e}↑+N_{e}↓}=A\tanh(t/\tau )(1+\Delta )$ as a function of *t*, the neutron TOF, here N↑ (*N*↓) are the measured proton and electron TOF spectra for neutron spin up and down. Low-energy electron events are selected for the *B* asymmetry measurement. For low-energy electrons, the proton recoils against the neutrino. The quantity *Δ* includes small corrections to polarization as discussed below. We show that *Δ*<2 × 10^−4^ and the uncertainty in *Δ* is less than 10^−4^. The neutron energy dependence of the measured asymmetries arises from the energy dependence of the polarization. Since B ≈ −1, less counting statistics are required for an accurate determination of the polarization than from A ≈ −1. The coefficient *B* and the combination of parameters *τ* can be determined without an auxiliary neutron polarization measurement by performing a two-parameter fit to the measured asymmetry as a function of TOF, *ε_B_* = *B* tanh(*t*/*τ*). Then *τ* for the same data set can be used to obtain *A*. This approach has the distinct advantage that the neutron polarization is determined by the same neutrons that are also used to determine *A* and *B*.

**Fig. 1:**
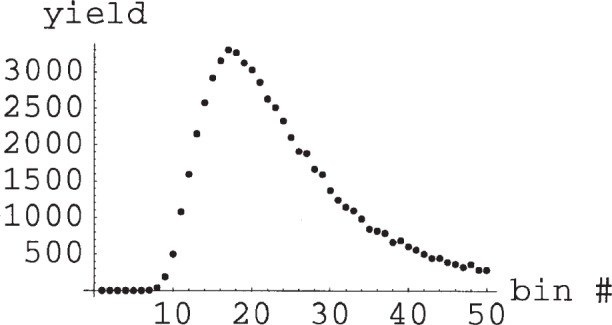
Yield per bin obtained in a 10 min run vs 1 ms wide time of flight bin for an assumed decay rate of 100 Hz for a 17 m long flight path.

**Fig. 2:**
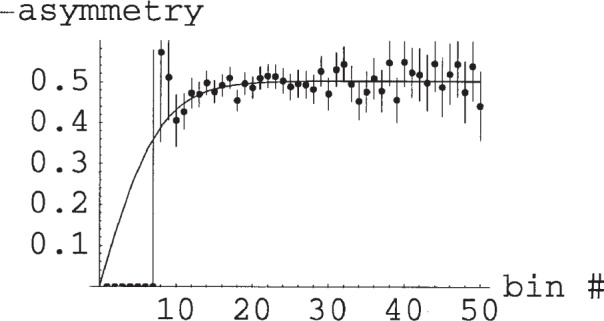
The neutron spin–neutrino momentum correlation coefficient, *B*, is determined from the proton asymmetry, *ε_B_* = (down−up)/(down+up), for low-energy protons. The proton recoils against the neutrino. In the pseudo-random data the coefficient *B* was assumed to be −1, the count rate 100 Hz, the cell thickness 7.5 bar-cm, and the ^3^He polarization 70 %. Asymmetry data and a two-parameter fit to *B* and the *τ* are shown. The asymmetry approaches −0.5 for long time of flight because the acceptance of each detector is 2π.

The approach is illustrated in Fig. 1, which shows the yield for a 10 min run *vs* TOF bin for an assumed decay rate of 100 Hz for a 17 m long flight path. In Fig. 2 we show pseudo-random asymmetry data generated for *P*_3_ = 0.7, cell thickness of 7.5 bar cm, and *B* = −1 over the time range from 0 ms to 50 ms. The fitted asymmetry is −0.993 ± 0.011, the fitted ^3^He polarization is 0.72 ± 0.11, and the fitted average neutron polarization is 0.989 ± 0.002. If the combination of parameters that determine *τ* were known *a priori* rather than determined from the two-parameter fit, the uncertainty of the asymmetry would be a factor of 0.79 smaller than from the fit. The price for the elimination of systematic errors is an increase in the statistical error by a factor of 1.27—a price well worth paying. The time-average neutron polarization is determined from the time dependence of the asymmetry signal and *B* is determined from the average asymmetry. In the abBA experiment, *B* will be determined from a large number of such runs. Although the neutron polarization has a statistical uncertainty of ≈1.5 % in each run, the asymmetry *B* can be determined to 0.1 % in ≈120 runs. The (uninteresting) time-average neutron polarization averaged over all runs would have an error of 0.0002. Although the neutron polarization in any given 10 min run is uncertain by 0.11 %, *B* can be determined with an uncertainty of 0.1 % by averaging over many runs. The determination of *A* will require 1.2 × 10^4^ 10 min runs or 6 × 10^6^ s. In the following sections we discuss various systematic uncertainties in the determination of the polarization and correlations.

## 2. Systematic Uncertainties From Time Drifts in the ^3^He Polarization

Drifts in the neutron polarization introduce corresponding drifts in the measured asymmetries *ε_A_* and *ε_B_*. The ^3^He polarization in the spin filter cell may change by 1 % in a few hours. The time scale is set by typical ^3^He polarization build-up and decay rates, which are intrinsically slow because the coupling of the ^3^He spin to its environment is weak. However, we seek to measure *B* (and *A*) ten times better than the typical drift. In order to understand how this can be done, first, consider a set of asymmetry data taken over a long period of time, *T*, with a constant ^3^He polarization. One could analyze the complete data set, or divide the data set into *N* subsets and analyze the *N* subsets separately and then average to obtain the asymmetry, *B*. The error in *B* for each subset would be increased by $\sqrt{N}$ but when the results are averaged over the *N* subsets, the value of *B* and the error in *B* would be the same as for the analysis of the complete data set. Next, consider a data set where the polarization is a slowly varying function of time. As the time span of subsets, *T/N*, approaches zero, the polarization may be taken to drift linearly. There is no loss in the statistical precision associated with subdividing the data. We estimate the shift in the asymmetry caused by the linear drift and show that the shift in *A* or *B* is negligible.

If the ^3^He polarization is constant, the neutron polarization as a function of *t* is given by *P*_n_(*t*) = tanh (*t*/*τ*). Assume that the ^3^He polarization is changing linearly with time within the run, *s*, ie., $P_{3}(s)=P_{3}+\frac{dP_{3}}{ds}s$. The neutron polarization as a function of TOF, *t*, and *s* is $P_{n}(t,s)=P_{n}(t)+\frac{dP_{n}(t)}{dP_{3}}\frac{dP_{3}}{ds}s+\frac{1}{2}\frac{d^{2}P_{n}(t)}{dP_{3}}{\left(\frac{dP_{3}}{ds}\right)}^{2}s^{2}+\ldots$ Next average this expression over the run time interval –*S* < *s* < *S* and insert the expression for the second derivative of polarization with respect to *P*_3_ to obtain the run average neutron polarization as a function of *t*. The second term gives zero because the average value of *s* is zero. The average of the third term gives a nonzero correction and is given by $E[\Delta P_{n}(t)]=\frac{t\;{\text{sech}}^{2}(t/\tau )[\tau -t\tanh(t/\tau )]}{\tau^{2}}{\left(\frac{dP_{3}}{ds}/P_{3}\right)}^{2}\frac{S^{2}}{3}+\ldots$ Note that, the correction term does not depend on whether the ^3^He polarization is decreasing or increasing with *s*. The largest value attained by the correction term is $0.6{\left(\frac{dP_{3}}{ds}/P_{3}\right)}^{2}\frac{S^{2}}{3}$ at *t* = 0.5*τ*. If we take *S* =5 min and $\frac{dP_{3}}{ds}=0.01$ per hour, the largest value of the correction term is 2.8 × 10^−7^. A change in the form neutron polarization versus *t* is completely negligible.

## 3. Inhomogeneous ^3^He Thickness

If the ^3^He cell length, *l*, is not equal across the neutron beam, then the relationship between the beam polarization and TOF is modified. Let *Q*(*l*) be the probability density function of cell thicknesses, *l*. Then

$$P_{n}=P_{n}(\bar{l})+\frac{1}{2}\frac{d^{2}P_{n}}{dl^{2}}\sigma_{l}^{2}+\ldots ,$$

where $\bar{l}=\int lQ(l)dl$, $\sigma_{l}^{2}=\int {\left(l-\bar{l}\right)}^{2}Q(l)dl$, and $\frac{1}{2}\frac{d^{2}P_{n}}{dl^{2}}=t^{2}/\tau \;\tanh\;(t/\tau )\;\text{sech}\;{\left(t/\tau \right)}^{2}/{\bar{l}}^{2}$. $P_{n}=\tanh\;(t/\tau )\left(1-{\left(\frac{t}{\tau }\right)}^{2}{\left(\frac{\sigma_{l}}{l}\right)}^{2}\right)$, where $\frac{\sigma_{l}}{l}$ is the fractional RMS variation in the thickness. For *P*_3_ = 0.7, *τ* = 6.90ms, and $\frac{\sigma_{l}}{l}=1\%$, the maximumvalue of the correction term is 4 × 10^−5^ at *t* = 9.3 ms. A small variation in the ^3^He thickness can be measured by observing the attenuation of a pencil beam of cold neutrons as the cell is scanned across the beam. This source of uncertainty is small and easily manageable.

## 4. Depolarization of the Neutron Spin in Material

After being polarized in the ^3^He cell the neutron spin can be depolarized when interacting with nuclei with non-zero-spin or with unpaired atomic electrons. This can take place when the neutrons passing through material like the exit wall of the ^3^He glass cell or aluminum windows of the RFSF or the entry window of the spectrometer. GE180 glass [2], used in the ^3^He spin filter cells, has several metal oxides, most of them as small residuals. The spin-flip cross sections (2/3 of the incoherent cross sections) were used to calculate the probabilities for the spin flips for a 3 mm thickness of GE180 glass. The largest spin-flip probability of 1.24 × 10^−4^ is for BaO_2_ which is 18.2 % by weight in the glass. The total spin-flip probability for the GE180 glass is 3.05 × 10^−4^. However, the neutrons are scattered into 4π and the solid angle of the decay volume is ≈4π × 10^−3^. The depolarization of the beam from nuclear scattering in the cell walls is thus ≈3 × 10^−7^ and is negligible. The spin-flip cross section of aluminum is 6.5 mb which gives a beam depolarization probability of ≈4 × 10^−7^ for a thickness of 1 cm and an acceptance of 4π × 10^−3^. GE180 has also a small amount of Fe_2_O_3_ component, 0.03 % by weight that can depolarize the neutron beam by spin-flip scattering by an unpaired electron. The total cross section for this spin flip is on the order of [4π(*γe*^2^/*m_e_c*^2^)] = 3.6 b [3] (*γ* = −1.91 is the gyromagnetic ratio of the neutron, *e* is the charge of the electron, and *m_e_c*^2^ is the electron mass), which gives a spin-flip scattering probability of order 6 × 10^−6^. The depolarization by scattering from un-paired electrons is negligible.

## 5. Depolarization in Inhomogeneous Magnetic Fields

We have studied neutron spin depolarization in inhomogeneous magnetic fields. The neutron beam is polarized by the ^3^He spin filter, then the beam passes through the adiabatic radio frequency spin flipper and finally enters the high-field decay region in the field-expansion spectrometer. The function of the RFSF is to provide a fast spin flip with efficiency very close to unity. It is necessary that the spin be transported from the RFSF to the decay region with negligible loss of polarization. Depolarization can take place when a neutron passes through the fringing field of the spectrometer and/or in the adiabatic RFSF. We have developed approximate expressions for the neutron spin wave function and the projection of the spin on the field as the neutron moves in space where the field direction and magnitude changes. The basic adiabaticity requirement is that the rate of a change of the field direction, has to be smaller compared to the Larmor frequency *ω* = *μB*/h (*μ* is the magnetic moment of the neutron) then the projection of the spin on the field direction is approximately conserved; the projection of the neutron spin on the field direction is an adiabatic invariant. In the design of the abBA experiment we will have a field configuration of $B=B_{0}x+\frac{dB}{dz}v\;tz$ where the *x*-axis is the axis of the spectrometer magnet and the *z*-axis is the beam axis, in two situations. First, the polarized neutrons must make transition through the zero in the vertical component of the spectrometer magnet when entering the spectrometer. We plan to apply an additional horizontal field that transports the spin adiabatically through the zero region. Second, we will flip the neutron spin using an adiabatic RF spin flipper. In such a device, the vertical field has a small gradient. The RF is applied horizontally at a frequency that matches the Larmor frequency at some point along the neutron trajectory. In the Larmor frame, the neutron spin adiabatically follows the combination of the RF and vertical field. A perturbative solution for the spin wave function in the coordinate system with the magnetic field in the *z* direction yields an asymptotic form for the depolarization, *D* = (*π*/6) exp [(4/3)−(*πλ*/2)], where the adiabaticity parameter *λ* is

$$\lambda =\frac{\mu B_{0}^{2}}{2\hbar \frac{dB}{dz}v}=\frac{\omega_{1}B_{0}}{2\frac{dB}{dz}v},$$

and *ω*_1_/*B* = 1.8 × 10^8^ rad s^−1^ T^−1^ (1.8 × 10^4^ rad s^−1^ G^−1^) is the angular precession rate of the neutron spin in a magnetic field. The gradient in the vertical field for our magnet design is ≈0.2 T/cm (2 × 10^3^ G/cm). A 10 meV neutron has a velocity of ≈1.4 × 10^5^ cm/s. Solving *λ* = 6 or *D* = 1.6 × 10^4^ gives *B*_0_ = 0.043 T (0.43 kG) for a horizontal guide field in the neighborhood of the vertical field zero. A practical RF field strength is a few tenths of a mT (a few G). For *B*_RF_ = 0.5 mT (5 G), we obtain a field of d*B*/d*z* = 0.027 mT/cm (0.27 G/cm). Neither of these numbers presents difficulty.

## 6. Neutron Energy Calibration and Intrinsic Neutron Pulse Width

In addition to the proton pulse width of about 1 μs at the SNS, further time broadening is introduced by the neutron moderation process. In a simplified model a shape of a cold neutron pulse for a single neutron energy is a Maxwell-Boltzmann TOF distribution. The width of the pulse is about 250 μs with a 600 μs tail. A number of the neutrons in the 600 μs tail has a 1/*E* dependence. The width of the neutron pulse has to be considered when determining the neutron energy by a TOF measurement. A 5 meV neutron has a 17 ms TOF for the 17 m long flight path. The first order shift in the TOF is ≈400 μs/17 ms ≈0.023. The moderation time can be measured *in situ* with a single crystal that scatters a monochromatic portion of the neutron beam or using a single free running chopper [4]. TOF of monochromatic neutrons gives the time profile for moderation of the neutrons of this energy. The moderation time profile will be measured for several neutron energies and the results fitted by adjusting parameters needed to describe the moderator. For the above example, the first order correction to the average polarization is 2 × 10^−3^ and the second order correction is 1 × 10^−7^. A measurement of the TOF with an accuracy of 5 % will reduce the uncertainty of the polarization from the neutron moderation time to 10^−4^.
